# Trajectories of prolonged grief one to six years after a natural disaster

**DOI:** 10.1371/journal.pone.0209757

**Published:** 2018-12-21

**Authors:** Josefin Sveen, Kerstin Bergh Johannesson, Martin Cernvall, Filip K. Arnberg

**Affiliations:** 1 National Center for Disaster Psychiatry, Department of Neuroscience, Uppsala University, Uppsala, Sweden; 2 Palliative Research Centre, Department of Health Care Sciences, Ersta Sköndal Bräcke University College, Stockholm, Sweden; 3 Stress Research Institute, Stockholm University, Stockholm, Sweden; University of Zurich, SWITZERLAND

## Abstract

**Background:**

The long-term trajectories of prolonged grief are poorly understood. The aims were to examine the course of grief among bereaved disaster survivors up to six years post loss and factors predicting worse bereavement outcome. A third aim was to explore differences in grief indicators between trajectories.

**Methods:**

Bereaved Swedish tourists who survived the 2004 Indian Ocean tsunamis responded to surveys including the Inventory of Complicated Grief 1 to 6 years after the disaster. Latent growth mixture modeling was used to identify longitudinal trajectories of grief. Multinomial logistic regression analysis was used to examine predictors of class membership.

**Results:**

Three trajectories were identified: resilient (41% of the sample), recovering (48%), and chronic (11%). The strongest predictor of chronic grief was the loss of one’s child. When examining grief indicators, the chronic trajectory was characterized by not accepting the loss, while yearning was common in all trajectories.

**Conclusions:**

This study highlights the importance of considering how traumatically bereaved individuals can be affected by loss for several years after a disaster, especially after losing one’s child. An inability to accept the loss, more so than yearning, appears to characterize bereaved survivors at risk of a chronic trajectory of grief.

## Introduction

Even though bereavement is common in disasters [1], most studies of disaster survivors have focused on posttraumatic stress rather than grief. Traumatic loss can be more detrimental and harder to resolve than natural or less sudden losses [2, 3]. While the majority of bereaved individuals adjust to the loss of a loved one without professional help, a significant minority will experience persistent and intensive grief reactions, commonly termed prolonged grief [4, 5], which can persist for years [6, 7]. An intense and lasting yearning for the deceased is considered one of the core symptoms of prolonged grief disorder (PGD) [5]. According to the just released ICD-11, PGD is also characterized by intense emotional pain such as difficulty accepting the loss and an inability to experience positive mood. In order to meet criteria for PGD, these reactions must also be associated with functional impairment and be present at sufficiently high levels for at least six months after the death [8]. In the DSM-5, Persistent Complex Bereavement Disorder is included as a condition for further study and symptoms should be persistent for at least 12 months in bereaved adults and 6 months for bereaved children and adolescents [9]. PGD is distinct from other mental health disorders, such as posttraumatic stress disorder and depression, but with considerable comorbidity [10, 11]. A recent meta-analysis, including studies up until 2016 of natural bereavement with time since loss ranging from 6 months to 12 years, reported a pooled prevalence rate of PGD to 9.8% (95% CI 6.8–14.0) [12].

High rates of PGD, approximately 40–50%, are reported among those who have experienced traumatic loss after a disaster [13–16]. Female gender and loss of close relatives, especially loss of children, have been found to be predictors of PGD after a disaster [14, 15, 17, 18]. One study found that direct exposure to a disaster was not associated with higher rates of PGD compared to disaster-bereaved individuals who were not exposed [14], while another study did find that exposure was associated with PGD [18]. Other factors that have been found to increase psychological distress among disaster-bereaved individuals are lack of social support [19, 20] and multiple losses [21, 22]. Yet, a recent study did not find multiple losses to be a predictor of psychological distress after loss in disaster [23].

There is a lack of longitudinal studies that examine distinct patterns of grief in disaster-bereaved individuals and factors predicting the different trajectories. To date, two studies [24, 25] have examined different patterns of problematic grief over time after the loss due to natural causes. Djelantik et al. [24] found four trajectories of PGD symptoms in bereaved individuals, using two time points, 6 and 18 months post loss: a persistent high PGD symptom-trajectory, a persistent moderate, a decreasing moderate and a persistent low PGD symptom-trajectory. In the study, the majority (90%) of individuals had lost a loved one due to a natural cause. In a similar study with one additional assessment at 12 months, Nam [25] identified two trajectories, a persistent high and a persistent low grief trajectory, in a sample of individuals who had lost a relative in dementia, mainly spouses.

The 2004 earthquake in the Indian Ocean and the subsequent tsunamis devastated coastal regions in Southeast Asia and more than 227,000 people perished [26]. At the time, an estimated 7,000 Swedish citizens were travelling in Southeast Asia in the areas hit hardest by the tsunamis. Previous studies have shown that one year to three years post-disaster traumatic bereavement had a considerable impact on psychological distress among the Swedish disaster survivors [18, 27]. There are studies from other disasters showing that traumatic bereavement can lead to high distress levels which persists for several years [23, 28]. As bereavement interventions should target those with persistent and prolonged distress [29], it is important to identify individuals who are at risk and in need of support. To our knowledge, there are no long-term longitudinal studies on trajectories of grief after a natural disaster. This study aimed to examine distinct patterns of grief among bereaved disaster survivors up to six years post loss. A second aim was to investigate factors predicting the different trajectories. A third aim was to explore differences in grief indicators between the distinct trajectories.

## Materials and methods

### Procedure and participants

Swedish authorities registered Swedish citizens returning from destinations in Southeast Asia at national airports during the three weeks after the 2004 earthquake in the Indian Ocean. Individuals 16 years of age or older (*n* = 10,501; 77% of those registered) were invited to participate in a postal survey approximately 1 year (14 months) after the disaster (T1). Of those, 4,932 people responded. There were 385 people who actively declined participation, mainly stating their reason being that they were not at all exposed to the disaster. Among the respondents at T1, 475 individuals had lost a relative. The respondents at T1 (*n* = 4,932; 49% response rate) were invited to participate in a second survey approximately three years (T2) and, regardless of the response at T2 they were invited to the third survey six years (T3) after the disaster. At T2, 3,457 (70%) responded whereof 132 individuals had lost a relative, and at T3, 2,643 (53%) responded, whereof 119 had lost a relative. This study includes 170 individuals with direct exposure to the tsunami and who lost a relative, including family members, in the tsunami and who filled in the Inventory of Complicated Grief (ICG) in at least one of the surveys. Of those, 166 individuals completed the ICG at T1, 122 at T2 and 102 at T3. In total, 88 individuals completed the ICG in all three surveys.

Descriptive data for the sample are presented in Table 1. Of the 170 individuals who lost a relative in the disaster, 50 individuals lost children, 46 lost a spouse/partner, 6 lost a girl/boyfriend, 54 lost parents, 25 lost siblings, 6 lost grandparents, 20 lost a parent-in-law, and 36 lost other relatives. Fifty-two individuals lost more than one relative.

**Table 1:** Sample characteristics and mean scores on Inventory of Complicated Grief.

	Sample (*n* = 170)	
	**Frequency**	**Percentage**
Gender		
Female	104	61%
Male	66	39%
Age at time of study (years)		
16–24	25	15%
25–40	55	32%
41–60	79	47%
61+	11	6%
Family situation		
Married/partner	96	57%
No partner	74	43%
Level of education		
≤ 12 years	89 (78^§^)	52%
> 12 years	80	48%
Pre-disaster depressive/anxiety problems (missing n = 60; 35%)		
Yes	20	12%
No	90	53%
Post-disaster adverse events up to T1		
≥3	9	
1–2	41	
0	120	
	**Mean (Range)**	**SD**
IES-R total at T1 (n = 169)	36.44 (1.00–82.00)	18.89
Satisfaction with social support at T1 (n = 168)	5.19 (1.00–7.00)	1.65
ICG total sum at T1 (n = 166)	34.58 (2.10–66.50)	15.48
ICG total sum at T2 (n = 122)	29.38 (0.00–59.00)	15.07
ICG total sum at T3 (n = 102)	24.64 (0.00–55.00)	14.28

IES-R:Impact of Event Scale-Revised; ICG:Inventory of Complicated Grief

^§^ 11 individuals were ≤ 20 years, and were not old enough to have completed 12 years or more of education.

Of those invited to participate in T1 (N = 10,501), which includes bereaved and non-bereaved individuals, younger age and male sex was associated with a slightly lower probability of responding [13]. Bereaved participants at T2 were compared with bereaved participants lost to follow-up at T2 regarding gender, age, marital status, posttraumatic stress, general mental health, and prolonged grief reactions at T1. The same comparison was conducted for T3, and the only differences found were that participants at T3 had higher scores on general mental health problems assessed with the General Health Questionnaire 12 [30] at T1, *t*(167) = -2.2, *p* = .031, and were older than those lost to follow-up at T3, χ^2^ = 12.1, *p* = .007. The study was approved by the Regional Ethics Review Board in Uppsala, Sweden (Reg. no. 2005:157; 2010/412)

### Measures

#### Survey instrument

Questions regarding participants’ demographics, disaster exposure, and adverse life events post-tsunami were included in the survey. Gender, family situation (*married/partner* or *no partner*) and education level (≤12 or >12 years, i.e. higher education) were coded as dichotomous variables.

Disaster exposure severity was categorized into two direct exposure groups, severe and moderate exposure, and one indirect exposure group (not included in this study), according to a set of 30 yes/no questions [31]. The items included frequently used exposure criteria but were tailor-made to the tsunami according to outcome, culture, place, type of event, and appraisal or significance of the disaster. The severe exposure group included participants who reported exposure to life threat or who were caught or close to being caught in the tsunami. The moderate exposure group included individuals who indicated “No, I was not in the area of being caught by the tsunami wave”, but who reported one or more of the following: loss of relatives, subjectively felt a threat to life, physical injury to themselves or others, anxiety regarding the fate of relatives, helped other victims, or witnessed corpses, others’ suffering, or forlorn children.

Satisfaction with social support was assessed at T1 with one item out of seven from the Crisis Support Scale: “*Overall*, *are you satisfied with the social support you have received after the tsunami*?” (Joseph *et al*., 1992). Respondents rated their overall satisfaction on a seven-point Likert scale ranging from 1 (*never*) to 7 (*always*). This item has previously been analyzed separately from the other items [32–34].

Adverse events were assessed at T1 with a checklist of 13 items [35]. Respondents were asked to indicate whether they had experienced post-disaster stressful events not related to the tsunami, including accidents, disasters, war/terror, violence/abuse, severe illness/injury to self or relatives, severe family conflicts/divorce, parents’ divorce, and death of a significant other. The responses were coded into three categories (*0*, *1–2*, and *≥ 3* events) [31].

Pre-disaster depressive and/or anxiety problems were screened for with two items developed by the research group: (a) *Before the tsunami disaster*, *did you ever feel depressed*, *in a low mood*, *or have feelings of hopelessness for more than two weeks*? (b) *Before the tsunami disaster*, *did you ever have problems with panic reactions*, *persistent anxiety or anguish for more than four weeks*? If participants answered yes, then they were asked to report whether any of these problems impacted their work or social functioning, or whether they had received psychological or pharmacological treatment for the problems. If the participant indicated either functional impairment or treatment for depressive or anxiety problems, previous depressive/anxiety problems were coded as present.

The Impact of Event Scale–Revised (IES-R) [36] was used to assess post-traumatic stress (PTS) symptoms at T1-T3, and the T1 assessment was used in this study. The questionnaire contains 22 items comprising three subscales: intrusion (8 items), avoidance (8 items), and hyperarousal (6 items). The items are keyed to a specific event in the past, which in this case was the tsunami. The respondents rated how distressing these reactions had been during the past seven days on a five-point Likert scale ranging from 0 (*not at all*) to 4 (*extremely*), yielding a total score of 0-88. Cronbach’s alpha value at T1 was .95. A psychometric study of a subset of the Swedish tsunami cohort indicated excellent temporal stability of the factor structure of the IES-R [37].

The Inventory of Complicated Grief (ICG) [38] was used to assess symptoms of complicated grief at T1-T3. The ICG comprises 19 items, including yearning for the deceased, preoccupation with the deceased that interrupts normal activities, trouble accepting the loss, detachment, bitterness, loneliness, feeling that part of one’s self died, feeling that life is empty, and loss of security or safety. The respondents rated the frequency of symptoms during the past month on a 5-point scale (0 = *never*, 1 = *rarely*, 2 = *sometimes*, 3 = *often*, 4 = *always*). In the first survey, item number 18 (“*I hear the voice of the person who died speak to me”*) was omitted due to a technical error; thus imputation was made by taking the average of the 18 items and adding to the total sum. Internal consistency was high across the three time points; Cronbach’s α values ranged from .92 to .93 and mean Inter-Item correlations ranged from .40 to .43.

### Statistical analysis

Preparatory data analysis and multinomial regressions were performed using IBM SPSS version 22.0 for Windows. Demographic and outcome variables were checked for anomalies. At least 67% of the items in the IES-R and ICG had to be completed for the total score to be included in the study, and missing data were replaced with the individual’s mean for all the completed items. One participant had more than 33% missing items on the IES-R and one participant had more than 33% missing on ICG.

Latent growth mixture modeling (LGMM) using MPlus 8 software [39] was used to identify longitudinal trajectories of grief. In the LGMM analysis, one to five classes were estimated and compared based on the interpretability of the model, the number of participants in the classes and a set of indices commonly used to assess model fit: Akaike’s Information Criterion (AIC), Bayesian Information Criterion (BIC), Sample Size Adjusted BIC (SSA-BIC), for which lower values indicate better model fit; Lo-Mendell-Rubin likelihood ratio test (LMR LRT) and the bootstrapped LRT (BLRT), which indicate whether adding a class to the model is associated with statistically significant improvement in model fit; and entropy, a summary measure between 0 and 1 of the classification accuracy and a value closer to one indicate better accuracy [39]. Although there are no established cut-off criteria for acceptable levels of entropy, Clark and Muthén [40] give guides of 0.4, 0.6, and 0.8 as representing low, medium, and high entropy, respectively. Good model fit was indicated by the lowest BIC value and the highest p-value of Bootstrap LMR [41]. The LGMM utilizes a robust full information maximum-likelihood (FIML) estimation procedure and under the assumption that missing data are missing at random, it provides unbiased estimates using all available observations. As a sensitivity analysis, the LGMM analysis was replicated including only the completers. After the model with the best fit was determined, multinomial logistic regression analyses were used to examine potential predictors of class membership, including age, gender, exposure severity, loss of child, multiple loss, stressful life events after the trauma, symptom of PTS at T1, social support at T1. First the predictors were entered one by one (bivariate regression) and then all the predictors were entered at the same time. The multinomial regression compares each group with a reference category without assuming any order between categories. Marital status was not included in the regression analysis as 51 participants had lost a partner. Four participants were removed from the multinomial regression due to missing values on the predictors.

The grief indicators yearning, disbelief, anger and acceptance of death were assessed by using single items from the ICG as used in the study by Maciejewski et al. [42]. Analysis of variance (ANOVA) and Bonferroni correction were used to examine the statistical significance of the differences in the grief indicators across the latent classes. Pearson’s correlations were used to assess associations between the indicators and one item from the ICG concerning functioning.

## Results

**Fig 1:**
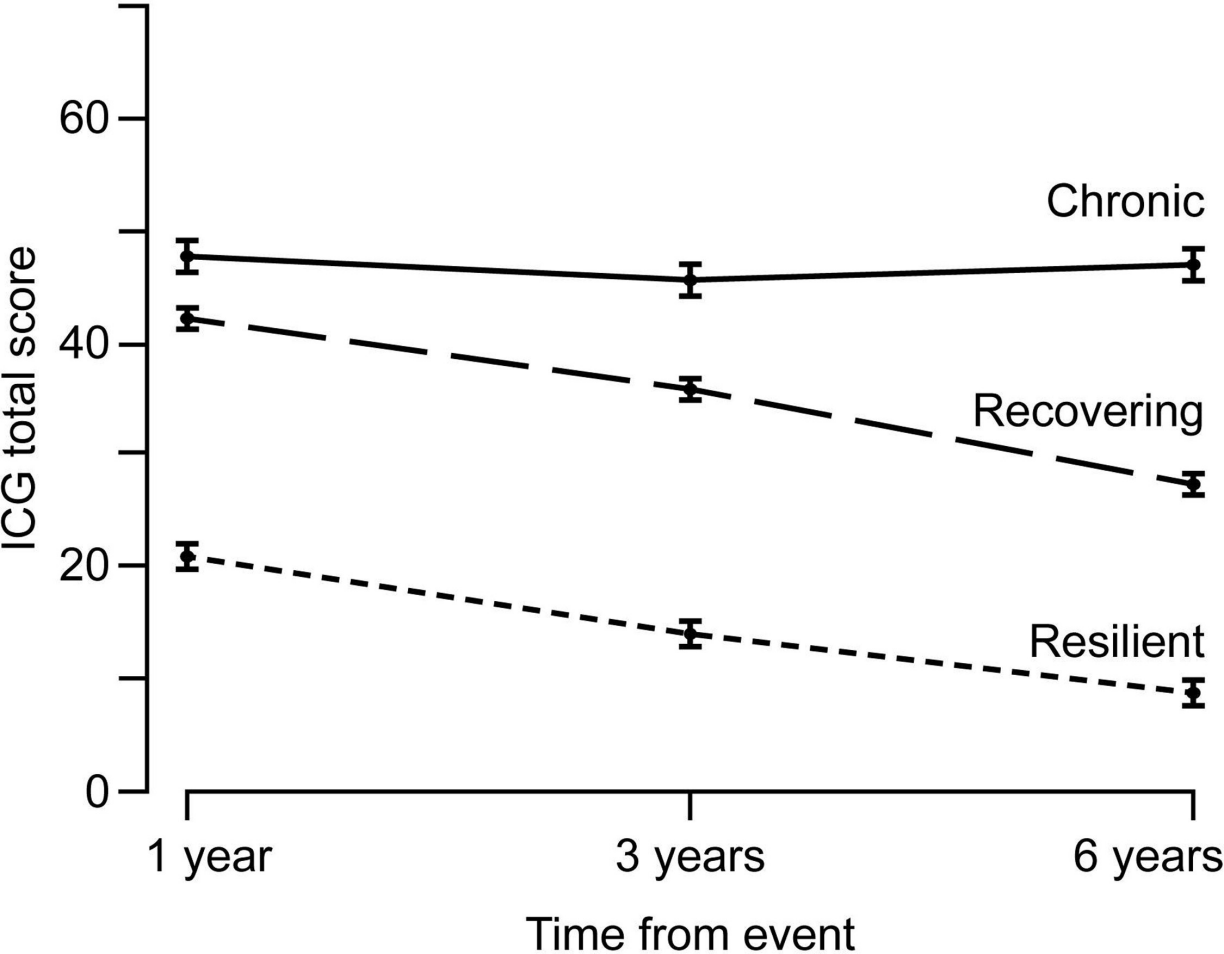
Trajectories of grief among bereaved disaster survivors. Note: ICG, Inventory of Complicated Grief. Error bars indicate standard errors.

In the latent growth mixture models, the entropy was acceptable for all models and the model fit for three and four-class solutions were moderately similar. The three-class model had a better fit according to the BIC and the Bootstrapped LMR LRT (Table 2). In addition, the three-class model emerged to have better interpretability and parsimony than the other models. Thus, the three-class model was chosen for further analyses. This model identified three distinct trajectories of grief symptoms from 1 to 6 years after the traumatic loss (Fig 1).

**Table 2:** Fit indices for one to five-class unconditional latent growth mixture models of long-term grief (ICG) in bereaved disaster survivors.

Number of classes	AIC^a^	BIC^b^	SSA-BIC^c^	Entropy	LMR LR^d^ *p*-value	Bootstrapped LRT *p*-value
One	2996.753	3021.840	2996.509			
Two	2986.475	3020.969	2986.139	0.703	0.007	0.0000
Three	2978.914	3022.816	2978.487	0.714	0.0956	0.050
Four	2973.845	3027.154	2973.326	0.754	0.1481	0.0698
Five	2978.722	3041.438	2978.111	0.705	0.8564	1.000

^a^Akaike’s Information Criterion.

^b^Bayesian Information Criterion.

^c^Sample Size Adjusted BIC.

^d^Lo-Mendell-Rubin likelihood ratio test.

The class labelled *resilient* (n = 70; 41%) was characterized by moderately low levels of prolonged grief at T1 with a monotonic decline through T2 to T3 (Intercept = 20.10; Slope = -2.39; *p* < .001). The moderate *recovering* class (n = 81; 48%) was characterized by a trajectory with initially high symptoms of prolonged grief and a decrease in symptoms thereafter (Intercept = 41.84; Slope = -2.84; *p* < .001). The class labelled *chronic* included 19 participants (11%) and was characterized by a trajectory with high levels of prolonged grief symptoms at each time point (Intercept = 46.83; Slope = 0.04; *p* = .94). The sensitivity analysis using only participants with ICG scores at all assessments verified these results (see S1 and S2 Supporting informations).

**Fig 2:**
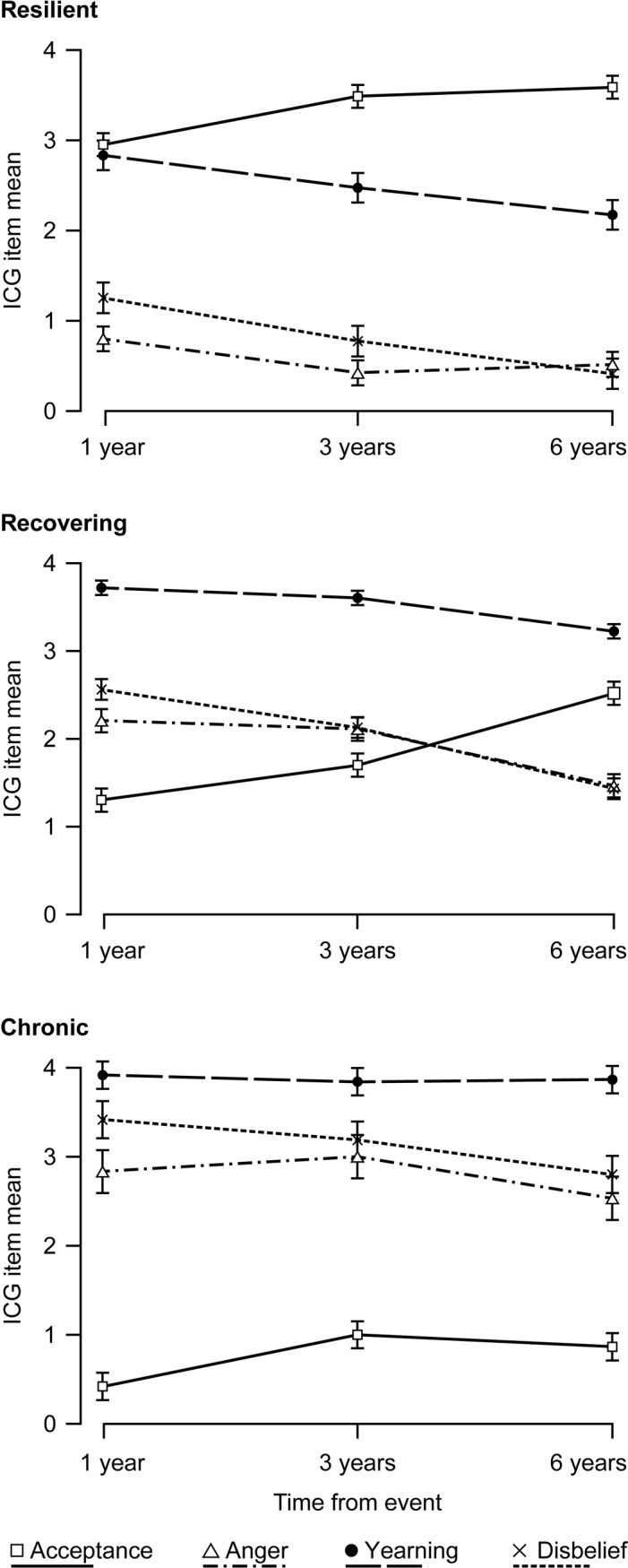
Characteristics of grief indicators in the distinct trajectories. Note: Error bars indicate standard errors; ICG, Inventory of Complicated Grief.

The multinomial regression analysis of potential predictors for class membership is shown in Table 3. The resilient group was chosen as the reference category against which the recovering and chronic groups were compared. The recovering group and the chronic group had a higher proportion of females. Participants in the recovering and chronic groups were more likely to have lost a child, but they were not more likely to have experienced multiple losses. The coefficients for loss of a child were nominally larger in multinomial models compared to bivariate models. The participants in the recovering and chronic groups had higher levels of PTS symptoms at T1 compared to the resilient group. Individuals in the recovering group were more likely to be satisfied with social support during the first year after the tsunami. There were no differences in exposure severity, age, or post-disaster events among the grief trajectories. The association between exposure severity and group membership was attenuated in the multinomial models compared to the bivariate models, especially for the resilience group, and this is likely due to the inclusion of posttraumatic stress reactions (IES) at T1 in multinomial models. To further explore the characteristics of grief in the distinct trajectory groups, they were examined with regards to grief indicators (Fig 2). The individuals in the chronic trajectory were characterized by not accepting the loss, even at T3. Moderate to high scores on the yearning item were present in all grief trajectories.

**Table 3:** Regression analysis of predictors for the prolonged grief trajectories, with the resilient class as the reference group.

	Recovering		Chronic	
Variable	Bivariate Odds ratio (95% CI)	Multinomial Odds ratio (95% CI)	Bivariate Odds ratio (95% CI)	Multinomial Odds ratio (95% CI)
Exposure severity				
High	3.47 (1.52–7.96)**	0.97 (0.30–3.11)	1.84 (0.55–6.16)	0.57 (0.10–3.26)
Moderate (ref)	-	-	-	-
Age at T1	0.99 (0.96–1.01)	1.0 (0.96–1.03)	1.01 (0.98–1.05)	1.01 (0.96–1.07)
Gender				
Female	2.24 (1.16–4.34)**	3.13 (1.22–8.00)*	3.97 (1.20–13.16)*	5.61 (1.23–25.52)*
Male (ref)	-	-	-	-
Post-disaster adverse events up to T1	1.19 (0.90–1.56)	1.17 (0.88–1.56)	0.73 (0.35–1.52)	0.54 (0.21–1.40)
Satisfaction with social support at T1	0.68 (0.54–0.85)***	0.74 (0.54–1.01)	0.68 (0.56–1.10)	0.83 (0.52–1.31)
IES-R at T1	1.10 (1.07–1.13)***	1.08 (1.05–1.11)***	1.12 (1.06–1.15)***	1.12 (1.07–1.17)***
Loss of child				
Yes	2.41 (1.11–5.24)*	3.56 (1.24–10.19)*	6.65 (2.21–20.02)***	14.00 (3.14–62.39)***
No (ref)	-	-	-	-
Multiple loss				
Yes	2.16 (1.06–4.42)*	1.55 (0.62–3.92)	0.88 (0.26–3.04)	0.61 (0.14–2.72)
No (ref)	-	-	-	-

CI, confidence interval; reference group = resilient trajectory (n = 67); recovering trajectory n = 81; chronic trajectory n = 18.

*p<0.05.

**p<0.01.

***p<0.001.

The resilient class had significantly higher levels of acceptance and lower levels of yearning, anger and disbelief at each time point than the recovering and chronic trajectories (Table 4). The recovering class had significantly higher levels of acceptance at T3 and lower disbelief at T1, T3 than the chronic trajectory, and lower levels of anger at T2 and T3 (Table 4).

**Table 4:** Differences between trajectories in grief indicators.

	F-value (*df*)	Post hoc*
**T1**		
Acceptance	44.63 _(2,163)_	1>2,3
Yearning	21.44_(2,163)_	1<2, 3
Disbelief	32.32_(2,162)_	1<2<3^a^
Anger	27.29 _(2,163)_	1<2, 3
**T2**		
Acceptance	58.23 _(2,118)_	1>2,3
Yearning	29.42 _(2,119)_	1<2, 3
Disbelief	40.89_(2,116)_	1<2,3
Anger	41.53 _(2,119)_	1<2<3^a^
**T3**		
Acceptance	52.99_(2,99)_	1>2>3
Yearning	25.59_(2,99)_	1<2,3
Disbelief	52.27_(2,99)_	1<2<3
Anger	26.23_(2,99)_	1<2<3

F-values are significant at *p* < .001.

* Post Hoc analysis with Bonferroni correction, trajectory figures (1 = Resilience, 2 = Recovering, 3 = Chronic) indicate significant group differences at *p* < 0.001,

^a^ indicate *p* < 0.05.

Finally, to characterize the different grief indicators, their levels at T3 were correlated with the functioning item of the ICG in the total sample. Acceptance was strongly correlated (*r* = 0.62, *p* < .01) while yearning was moderately correlated (*r* = 0.35, *p* < .01) with functioning. Anger and disbelief also were strongly correlated with functioning (*r* = 0.50 and *r* = 0.55, respectively; *p*s < .01).

## Discussion

This is the first longitudinal study of trajectories of grief following bereavement in a natural disaster. We found that a three-class model best represented the data. Although the entropy was at an acceptable level this model left substantial portions of the variation unexplained. The classes included a resilient trajectory comprising 41% of the sample characterized by moderately low levels of prolonged grief from one to six years post loss; a recovering trajectory including half of the individuals with initially high symptoms of prolonged grief and a gradual decrease in symptoms thereafter, and a chronic trajectory including 11% of the participants with high and unremitting levels of prolonged grief symptoms for as long as six years after the event. Here we note the caveat that the first assessment took place 14 months after the loss, and there were likely fluctuations in prolonged grief during the first year. The pattern of trajectories is in accordance with other studies as well as the percentage of individuals in the chronic trajectory. However, the resilient trajectory was not the most common response as commonly found. A possible explanation for the lower proportion of individuals in the resilient trajectory found in this study is that participants had been traumatically bereaved as well as directly exposed to the disaster, whereas other studies have included less traumatic loss and individuals who were not exposed to trauma.

In the analysis of potential predictors of class membership, the strongest predictor for prolonged grief was the loss of one’s child. Studies have found that the loss of a child is more detrimental than other types of loss and it is a known risk factor for PGD [43, 44]. One reason for that the death of child is more detrimental than other types of bereavement is the nature of the parent-child bonding and the rupture of attachment [45]. However, a recent study of trajectories of depression following child and spousal bereavement did not find the loss of one’s child, compared to spousal bereavement, to be a strong predictor of depression [46]. Taken together, the type of loss seems more significant for prolonged grief specifically, rather than for depressive symptoms.

Another predictor was PTS at one year post loss, which could be an indicator of the combined burden of exposure to a traumatic event and a traumatic loss, as well as the comorbidity between prolonged grief and PTS. Again, it is important to note that we did not measure grief or psychopathology during the first year, as high levels of distress during this period may have influenced both posttraumatic stress at one year and the chronic grief trajectories. Multiple losses were not a predictor of a more chronic trajectory in this study. These results suggest that the number of losses does not have an additive effect on chronic grief reactions; rather, it is the relation to the person lost that is important, for example the loss of a child, that is predictive of long-lasting grief.

When exploring the grief indicators, the chronic trajectory was characterized by low levels of acceptance of the loss even after six years, whereas the other trajectories had higher levels of acceptance. Endorsement of yearning was the most common of the grief indicators. Interestingly, acceptance had the strongest association with the ICG item on everyday functioning whereas yearning had the weakest association. Taken together, these results suggest that of the four grief indicators, lack of acceptance plays an important role in unresolved grief. This is consistent with previous studies suggesting that resolution of grief concurs with increasing acceptance of loss [47] and that acceptance of loss is associated with resilience [48]. Consistent with the current study, a recent study [49] found a high probability of yearning in both symptomatic and resilient subgroups of bereaved. Yet, a weakness of the present study is that the grief indicators were only measured with single items, taken from the ICG instrument, and not measured with several items on the same phenomena. However, the ICG has been validated and the items should be representative of the grief indicators used in the present study. Furthermore, these items have previously been used as grief indicators in a study by Maciejewski et al. [42].

One limitation of this study is the response rate, which was 49% in the first survey, which limits the study generalizability. Participation rates has generally decreased in longitudinal survey studies and similar rates are often reported [50]. A study on nonparticipants of a postdisaster survey found that nonparticipations was related to low exposure and lack of interest or time, while participation was related to PTS symptoms [51]. We cannot rule out that the low percentage of individuals in the resilient trajectory is due to resilient individuals not participating in the study. However, in a previous study on trajectories of PTS following the tsunami based on the same cohort but also including non-bereaved participants, thus a much larger sample (n = 2,268), the resilience trajectory of PTS was large, 72% [52]. These previous results suggest that resilient individuals are participating in the current study. Furthermore, loss to follow up may have affected the trajectories, however, the LGMM uses a FIML estimation procedure and the participants that dropped out were similar to those retained in the study. Another limitation is that symptoms of prolonged grief were assessed with a self-report instrument and not by clinical interviews including an assessment of functional disability; thus, the correspondence of self-reported strong grief reactions to psychopathology is unclear. Nevertheless, the self-report instrument ICG has been shown to be a psychometrically sound scale to assess prolonged grief [38]. It could be considered a limitation that the first assessment was at one year after the disaster; hence we lack information about the initial grief reactions. However, the focus of this study was on prolonged grief which is defined as a protracted response to loss and according to DSM-5 the time criterion is that the symptoms should persist to at least one year post loss [9]. Finally, a limitation is the small sample size and resulting large confidence intervals in the regression model.

A strength of the study is the longitudinal design with follow-ups at up to six years post-disaster, which is very rare in grief research. The included sample is not representative of the general population in Sweden, as these individuals were tourists travelling to Southeast Asia and thus had higher socioeconomic status [53]. This fact, however, together with the nature of the disaster itself as a distant event and the participants’ subsequent return to unaffected communities conferred the benefit that the sample was less exposed to secondary stressors and additional stressful circumstances that could potentially negatively affect the specificity of the grief trajectories.

## Conclusions

This longitudinal study found prolonged grief among bereaved survivors from a natural disaster follow three trajectories, with a recovering trajectory being the most common. However, the entropy of the models indicates wide variations of grief reactions within these trajectories and it could be that the trajectories are too simplified to efficiently describe these variations. The loss of a child and posttraumatic stress symptoms were strong predictors of a worse long-term outcome. In addition, the results suggest that yearning is a common grief reaction that persists over time, while not accepting the loss is associated with unresolved grief. This study highlights the importance of considering how traumatically bereaved individuals can be severely affected by loss for several years after the disaster and that they are in need of additional support to promote healthy grieving.

## Supporting information

S1 Supporting information Fit indices for one to five-class unconditional latent growth mixture models of long-term grief (ICG) in bereaved disaster survivors including only completers (n = 88). (PDF)

S2 Supporting information Trajectories of grief among bereaved disaster survivors including only completers (n = 88). Note: ICG, Inventory of Complicated Grief. Error bars indicate standard errors. Resilient: n = 27 (31%); Recovering: n = 48 (53%), Chronic: n = 14 (16%).
(TIF)

S3 Supporting information Survey questions in Swedish and English. (PDF)
