# Supplementary material for: Trajectories of prolonged grief one to six years after a natural disaster
Source: PLoS One. 2018 Dec 21;13(12):e0209757. doi: 10.1371/journal.pone.0209757 (PMC6303052; doi:10.1371/journal.pone.0209757)
Supplement: S1 Supporting information — (PDF) [file pone.0209757.s001.pdf]

S1. Supporting information. Fit indices for one to five-class unconditional latent growth mixture models of long-term grief (ICG) in bereaved disaster survivors including only completers (n=88).

Number of Classes	AIC[a]	BIC[b]	SSA-BIC[c]	Entropy	LMR LR[d] *p*-value	Bootstrapped LRT *p*-value
One	1987.336	2007.155	1981.910			
Two	1980.268	2007.519	1972.807	0.801	0.1079	0.0000
Three	1974.173	2008.856	1964.678	0.862	0.1069	0.1241
Four	1974.039	2016.154	1962.509	0.824	0.2378	0.4286
Five	1972.799	2022.345	1959.234	0.833	0.6242	1.000

[a]Akaike's Information Criterion; [b]Bayesian Information Criterion; [c]Sample Size Adjusted BIC; [d]Lo-Mendell-Rubin likelihood ratio test
