# Supplementary figures and images for: Trajectories of prolonged grief one to six years after a natural disaster
Source: PLoS One. 2018 Dec 21;13(12):e0209757. doi: 10.1371/journal.pone.0209757 (PMC6303052; doi:10.1371/journal.pone.0209757)

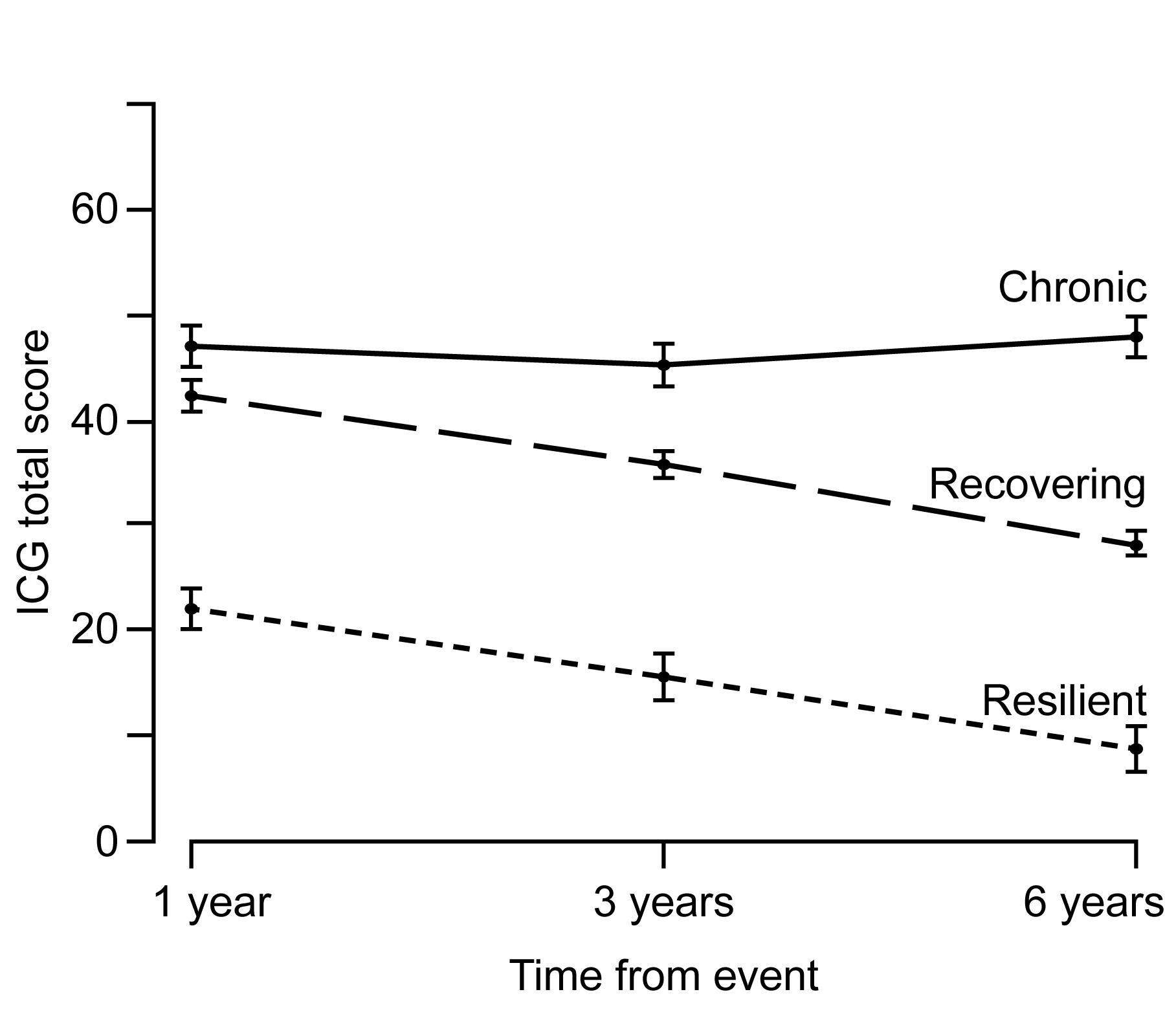

Supplement: S2 Supporting information — Note: ICG, Inventory of Complicated Grief. Error bars indicate standard errors. Resilient: n = 27 (31%); Recovering: n = 48 (53%), Chronic: n = 14 (16%). (TIF) [file pone.0209757.s002.tif]
