# Supplementary material for: Trajectories of prolonged grief one to six years after a natural disaster
Source: PLoS One. 2018 Dec 21;13(12):e0209757. doi: 10.1371/journal.pone.0209757 (PMC6303052; doi:10.1371/journal.pone.0209757)
Supplement: S3 Supporting information — (PDF) [file pone.0209757.s003.pdf]

Survey questions in Swedish

Impact of Event Scale-Revised

Här nedan hittar du en lista på svårigheter som människor ibland har efter svåra och stressande livshändelser. Läs varje påstående och ange sedan hur störande detta varit för dig under de senaste sju dagarna? Ett alternativ per rad

		Inte alls	Lite grann	Måttligt	Ganska mycket	Väldigt mycket
1.	Allt som påminner om Tsunamin väcker starka känslor	☐	☐	☐	☐	☐
2.	Jag har haft svårt att sova eller sovit oroligt	☐	☐	☐	☐	☐
3.	Saker och ting har påmint mig om Tsunamin	☐	☐	☐	☐	☐
4.	Jag har känt mig arg och lättretlig	☐	☐	☐	☐	☐
5.	Jag har försökt undvika att bli upprörd när jag har tänkt på Tsunamin eller blivit påmind om den	☐	☐	☐	☐	☐
6.	Jag har oavsiktligt kommit att tänka på Tsunamin	☐	☐	☐	☐	☐
7.	Tsunamin har känts overklig eller som om den inte har hänt	☐	☐	☐	☐	☐
8.	Jag har undvikit det som påminner om Tsunamin	☐	☐	☐	☐	☐
9.	Minnesbilder har dykt upp i huvudet	☐	☐	☐	☐	☐
10.	Jag har känt mig darrig och lättskrämd	☐	☐	☐	☐	☐
11.	Jag har försökt låta bli att tänka på vad som hänt	☐	☐	☐	☐	☐
12.	Jag är klar över att jag fortfarande har intensiva känslor, men jag har inte tagit itu med dem	☐	☐	☐	☐	☐
13.	Mina känslor för det som hänt känns som bedövade	☐	☐	☐	☐	☐
14.	Jag har kommit på mig själv att handla eller känna som om jag åter var tillbaka vid Tsunamin	☐	☐	☐	☐	☐
15.	Jag har haft problem att somna	☐	☐	☐	☐	☐
16.	Jag har överväldigats av starka känslor	☐	☐	☐	☐	☐
17.	Jag har försökt ta bort Tsunamin från minnet	☐	☐	☐	☐	☐
18.	Jag har haft svårt att koncentrera mig	☐	☐	☐	☐	☐
19.	Sådant som påminner mig om Tsunamin har givit mig kroppsliga symtom, t ex svettningar, andnings-svårigheter, illamående eller hjärtklappning	☐	☐	☐	☐	☐
20.	Jag har drömt om Tsunamin	☐	☐	☐	☐	☐
21.	Jag har känt mig vaksam och på min vakt	☐	☐	☐	☐	☐
22.	Jag har försökt låta bli att tala om Tsunamin	☐	☐	☐	☐	☐

Inventory of Complicated Grief

I vilken grad har du de sista två veckorna upplevt följande känslomässiga reaktioner? Ett alternativ per rad

		Aldrig	Sällan	Av och till	Ofta	Alltid
1.	Jag tänker så mycket på den/de döda att det är svårt för mig att göra saker som jag vanligtvis gör	☐	☐	☐	☐	☐
2.	Minnet av den/de döda gör mig orolig	☐	☐	☐	☐	☐
3.	Jag känner att jag inte kan acceptera att han/hon/de har dött	☐	☐	☐	☐	☐
4.	Jag längtar efter den/de döda	☐	☐	☐	☐	☐
5.	Jag dras mot platser och saker som jag associerar med den/de döda	☐	☐	☐	☐	☐
6.	Jag kan inte hjälpa att jag känner mig arg över hans/hennes/deras död	☐	☐	☐	☐	☐
7.	Jag har svårt att ta in att det har hänt	☐	☐	☐	☐	☐
8.	Ända sedan han/hon/de dog har jag haft svårt med att lita på andra	☐	☐	☐	☐	☐
9.	Ända sedan han/hon/de dog har jag känt att jag har tappat förmågan till att känna omsorg för andra människor eller jag känner avstånd till människor som jag tycker om	☐	☐	☐	☐	☐
10.	Jag har smärtor i kroppen	☐	☐	☐	☐	☐
11.	Jag tar omvägar för att undgå minnen om den/de döda	☐	☐	☐	☐	☐
12.	Jag känner att livet är tomt utan honom/henne/dem som dog	☐	☐	☐	☐	☐
13.	Jag hör rösten på den/de döda tala till mig	☐	☐	☐	☐	☐
14.	Jag ser den/de döda framför mig	☐	☐	☐	☐	☐
15.	Jag känner att det är orättvist att jag lever när han/hon/de dog	☐	☐	☐	☐	☐
16.	Jag känner mig bitter över hans/hennes/deras död	☐	☐	☐	☐	☐
17.	Jag avundas dem som inte har förlorat någon närstående	☐	☐	☐	☐	☐
18.	Jag känner mig ensam stora delar av tiden efter att han/hon/de har dött	☐	☐	☐	☐	☐
19.	Jag känner mig förvirrad över det som hände........	☐	☐	☐	☐	☐

SOCIALT STÖD

Här ber vi dig värdera det sociala stödet du har idag. Skalan är från 1-7, där 1 är aldrig och 7 är alltid. Ett alternativ per rad

	Aldrig 1	2	3	4	5	6	Alltid 7
Är du sammantaget nöjd med det sociala stöd du fått efter Tsunamin?	☐	☐	☐	☐	☐	☐	☐

LIVSHÄNDELSER

Nedan följer ett antal frågor om händelser som kan inträffa i en människas liv. Sätt ett kryss för varje händelse, utöver Tsunamikatastrofen, som du varit med om under olika perioder i ditt liv. Flera alternativ möjliga för varje påstående

**Typ av händelse**	Före 16 års ålder	16 års ålder – 25 dec 2004	Efter 26 dec 2004
Varit med om trafikolycka eller annan olycka	☐	☐	☐
Upplevt en naturkatastrof	☐	☐	☐
Upplevt krig eller terror	☐	☐	☐
Varit utsatt för allvarligt våld eller övergrepp	☐	☐	☐
Drabbats av allvarlig kroppslig sjukdom eller skada	☐	☐	☐
Drabbats av allvarlig kroppslig sjukdom eller skada hos familjemedlem	☐	☐	☐
Haft allvarlig konflikt med anhörig eller skilsmässa	☐	☐	☐
Föräldrarna skilda eller separerade	☐	☐	☐
Förälders död	☐	☐	☐
Syskons död	☐	☐	☐
Eget barns död	☐	☐	☐
Partners död	☐	☐	☐
Annan nära familjemedlems död	☐	☐	☐
Arbetslös mer än 1 månad	☐	☐	☐
Svåra ekonomiska problem	☐	☐	☐

Nu kommer några frågor som gäller din hälsa *före* tsunamikatastrofen.

1.a Har du någon gång i livet, före tsunamikatastrofen, känt dig nedstämd, deprimerad eller att det känts hopplöst, i två veckor eller längre? Ja Nej

1.b **Om ja**, var besvären så påtagliga att de påverkade ditt arbete och sociala liv negativt? Ja / Nej

1.c **Om ja**, fick du psykologisk eller medicinsk behandling för besvären? Ja / Nej

2.a Har du någon gång i livet, före tsunamikatastrofen, haft besvär med panikkänslor eller ihållande oro eller ångest, i fyra veckor eller längre? Ja / Nej

2.b **Om ja**, var besvären så påtagliga att de påverkade ditt arbete och sociala liv negativt? Ja / Nej

2.c **Om ja**, fick du psykologisk eller medicinsk behandling för besvären? Ja / Nej

Exponeringsfrågor:

2.1 Förlorade du familjemedlemmar, andra närstående eller vänner i Tsunamin?

☐ Ja

☐ Nej ⟶ Gå till fråga 2.3

2.2 Vem förlorade du? Om du har förlorat barn eller barnbarn ber vi dig också att uppge barnets/barnens födelseår. Använd 4 siffror**.** Flera alternativ möjliga

☐ Make/maka/sambo

☐ Pojk- flickvän (ej sambo)

☐ Barn Födelseår: Årtal ____ Årtal ____ Årtal ____ Årtal ____ Årtal ____

☐ Barnbarn Födelseår: Årtal ____ Årtal ____ Årtal ____ Årtal ____ ____

☐ Mor

☐ Far

☐ Syskon

☐ Mormor/morfar

☐ Farmor/farfar

☐ Svärföräldrar

☐ Andra släktingar

☐ Vänner/arbetskamrater

☐ Andra

4.1 Då Tsunamin kom, uppehöll du dig då på en plats som geografiskt drabbades av Tsunamin?

☐ Ja

☐ Nej ⟶ Gå till fråga 8.1

4.2 Var befann du dig då Tsunamin kom? Om du förflyttade dig under händelsen, använd tidpunkten då du blev uppmärksam på faran. Ange ett alternativ

☐ I båt, långt från land

☐ I vattnet nära stranden (t.ex. badade, snorklade, trampbåt) eller på/nära stranden

☐ På land en bit bort från stranden

☐ Annan plats

4.3 Ungefär hur många meter från den normala strandkanten befann du dig då Tsunamin kom?

____ meter

4.4 Ungefär hur många meter över normal havsnivå befann du dig då Tsunamin kom?

☐☐☐☐ meter

4.5 Uppehöll du dig inomhus då Tsunamin kom, och i så fall var?

☐ Nej, jag var utomhus
☐ Ja, på marknivå/första våningen
☐ Ja, på andra våningen
☐ Ja, på tredje våningen eller högre

4.6 Hade du ansvar för tillsyn av barn då Tsunamin kom?

☐ Nej, hade inte ansvar för barn
☐ Ja, jag hade ensam ansvar
☐ Ja, jag hade ansvar tillsammans med andra vuxna

4.7 Var befann sig dina närstående då Tsunamin kom? Flera alternativ möjliga

☐ Ej aktuellt, jag var ensam
☐ I båt, långt från land
☐ I vattnet nära stranden (t.ex. badade, snorklade, trampbåt) eller på/nära stranden
☐ På land en bit bort från stranden
☐ Annan plats

4.8 Märkte du skakningarna av jordskalvet på morgonen före Tsunamin?

☐ Ja
☐ Nej
☐ Osäker/Vet ej

4.9 Upptäckte du att vattnet drog sig tillbaka innan Tsunamin kom?

☐ Ja
☐ Nej → Gå till fråga 4.11
☐ Osäker/Vet ej → Gå till fråga 4.11

4.10 Hur reagerade du då vattnet försvann? Ange ett alternativ

☐ Jag stannade där jag var
☐ Jag rörde mig utåt för att titta närmare på naturfenomenet
☐ Jag rörde mig utåt för att hämta eller hjälpa någon
☐ Jag rörde mig utåt av andra skäl
☐ Jag rörde mig bort från stranden

4.11 Upplevde du situationen som livshotande för egen del när vågen kom?

☐ Ja
☐ Nej
☐ Vet ej

4.12 Hamnade du själv i vattnet? Ange ett alternativ

☐ Ja, jag förlorade all fysisk kontroll
☐ Ja, men jag bevarande delvis fysisk kontroll
☐ Nej, men jag var mycket nära att hamna i vattnet
☐ Nej, jag var inte i närheten av att hamna i vattnet

4.13 Var du vittne till att någon annan drogs med av Tsunamin?

☐ Ja
☐ Nej ⟶ Gå till fråga 4.15

4.14 Vilken/a relation/er hade du till denna/dessa personer? Flera alternativ möjliga

☐ Make/maka/sambo
☐ Pojk- flickvän (ej sambo)
☐ Barn
☐ Barnbarn
☐ Mor
☐ Far
☐ Syskon
☐ Mormor/morfar
☐ Farmor/farfar
☐ Svärföräldrar
☐ Andra släktingar
☐ Vänner/arbetskamrater
☐ Lokalbefolkningen som du kände
☐ Andra turister
☐ Andra

4.15 Upplevde du situationen som livshotande för dina närstående när vågen kom?

☐ Ej aktuellt, jag var ensam ⟶ Gå till fråga 6.1

☐ Ja
☐ Nej
☐ Vet ej

5.1 Tsunamin ledde för många till att man kom bort från sina närstående då Tsunamin drabbade. Vilka av följande alternativ stämmer bäst in på din situation?

- ☐ Vi var tillsammans då Tsunamin kom och klarade av att hålla ihop
- ☐ Vi var tillsammans då Tsunamin kom men kom sedan från varandra
- ☐ Vi var åtskilda men jag var säker på att mina närstående var i trygghet
- ☐ Vi var åtskilda och jag var osäker på om mina närstående var i fara
- ☐ Vi var åtskilda och jag var säker på att mina närstående var i fara

5.2 Om någon av dina närstående omkom: Hur lång tid tog det innan du insåg att de hade omkommit? Om fler än en, utgå från det dödsfall där ovissheten varade längst. Ange ett alternativ

- ☐ Ingen omkom eller är saknad → Gå till fråga 5.4

- ☐ Omedelbart
- ☐ Upp till 1 dygn
- ☐ Upp till 3 dygn
- ☐ Upp till 1 vecka
- ☐ Upp till 1 månad
- ☐ Mer än en månad

5.3 Om någon av dina närstående omkom: Hur lång tid tog det innan du officiellt fick bekräftat att de hade omkommit? Om fler än en, utgå från det dödsfall där ovissheten varade längst. Ange ett alternativ

- ☐ Omedelbart
- ☐ Upp till 1 dygn
- ☐ Upp till 3 dygn
- ☐ Upp till 1 vecka
- ☐ Upp till 1 månad
- ☐ 1 månad - 6 månader
- ☐ Mer än 6 månader
- ☐ Fortfarande obekräftat

5.4 Om du blev skild från någon av dina närstående som överlevde: Hur lång tid tog det innan du fick veta att de hade överlevt? Om fler än en, utgå från det dödsfall där ovissheten varade längst. Ange ett alternativ

- ☐ Ej aktuellt

- ☐ Upp till en timme
- ☐ Upp till 1 dygn
- ☐ Upp till 3 dygn
- ☐ Upp till 1 vecka
- ☐ Mer än 1 vecka

6.1 I vilken grad tror du följande omständigheter bidrog till att du överlevde? Ett alternativ för varje rad

1. Att du klarade av att uppfatta hur farlig situationen var
2. Att du blev varnad av andra
3. Att du klarade av att värdera olika handlingsmöjligheter
4. Din egen styrka/uthållighet
5. Din goda simförmåga/vattenvana
6. Att du klarade av att hålla dig fast i något
7. Att du hade tur att komma undan
8. Att du valde ut en plats som visade sig vara trygg
9. Att du hade kunskaper eller förmåga som du kunde använda
10. Att du samarbetade med andra i din närhet
11. Att du gjorde likadant som andra
12. Att du fick hjälp av andra
13. Att du fick skydd av träd, hus eller liknande
14. Bara tillfälligheter

6.2 Hjälpte du några andra?

☐ Ja

☐ Nej

☐ Osäker eller minns inte

7.2 Upplevde du någon av följande påfrestningar under de första dygnen sedan Tsunamin dragit sig tillbaka? Ett alternativ för varje rad

	**Var du vittne till något av följande?**	Nej	Ja
1.	Människor som letade efter sina närmaste	☐	☐
2.	Övergivna barn	☐	☐
3.	Överlevande med allvarliga kroppsskador	☐	☐
4.	Mängder med omkomna	☐	☐
5.	Ett fåtal omkomna	☐	☐

	**Ovisshet/Osäkerhet**	Nej	Ja
6.	Var du osäker på någon av dina närmastes öde	☐	☐
7.	Hörde du rykten om eller var du rädd för att det skulle komma nya vattenmassor	☐	☐
8.	Var du osäker på om du skulle stanna där du var eller förflytta dig	☐	☐
9.	Saknade du information	☐	☐

		Nej	Ja
10.	Saknade du möjlighet att meddela dig hem	☐	☐

Andra förhållanden

		Nej	Ja
11.	Behövde du själv hjälp på grund av att du var skadad	☐	☐
12.	Var en eller flera av dina närstående skadade och behövde hjälp	☐	☐
13.	Var det andra skadade i din närhet som behövde hjälp	☐	☐
14.	Saknade du nödvändiga mediciner eller läkarbehandling	☐	☐
15.	Saknade du mat eller vatten	☐	☐
16.	Saknade du kläder och/eller annan utrustning	☐	☐
17.	Var du rädd för giftiga ormar, mörker, brand eller annat	☐	☐

		Nej	Ja
18.	Hade du ansvar för egna eller andras barn	☐	☐
19.	Hade du problem med vidare evakuering (transportproblem)	☐	☐
20.	Hade du förlorat viktiga ägodelar (glasögon, mediciner och liknande)	☐	☐
21.	Annat	☐	☐

22. Om annat, beskriv: ____

____

7.3 Blev du kroppsligt skadad, och hur allvarliga var i så fall skadorna? Ange ett alternativ

☐ Ja, allvarliga skador

☐ Ja, lätta skador

☐ Nej, inga skador

7.4 Blev någon eller några av dina närstående skadade, och hur allvarliga var i så fall skadorna? Ange ett alternativ

☐ Ja, allvarliga skador

☐ Ja, lätta skador

☐ Nej, inga skador

General Health Questionnaire 12 kan inte reproduceras på grund av copywrights. Reference: Sconfienza, C. (1998). Mätning av psykiskt välbefinnande bland ungdomar i Sverige. Rapport: Arbete och Hälsa 1998:22 av Arbetslivsinstitutet.

Survey questions in English

Impact of Event Scale Revised is cited in: Weiss, D. S., & Marmar, C. R. (1996). The Impact of Event Scale - Revised. In J. Wilson & T. M. Keane (Eds.), *Assessing psychological trauma and PTSD* (pages 399-411). New York: Guilford.

Inventory of Complicated Grief is cited in: Prigerson, H. G. et al. (1995). Inventory of Complicated Grief: a scale to measure maladaptive symptoms of loss. Psychiatry Research, Vol 59, pages 65-79.

Crisis support scale is cited in: Joseph S, et al (1992). Crisis support and psychiatric symptomatology in adult survivors of the Jupiter cruise ship disaster. British Journal of Clinical Psychology. Vol. 31, pages 63-73

General Health Questionnaire 12 cannot be reproduced because of copywrights. Reference: Goldberg, D.P. (1972). The detection of psychiatric illness by questionnaire. London, Oxford University Press.

Pre-disaster depressive and/or anxiety problems cited in the article. a) *Before the tsunami disaster, did you ever feel depressed, in a low mood, or have feelings of hopelessness for more than two weeks*? (b) *Before the tsunami disaster, did you ever have problems with panic reactions, persistent anxiety or anguish for more than four weeks?* If participants answered yes, then they are asked to report whether any of these problems impacted their work or social functioning, or whether they have received psychological or pharmacological treatment for the problems. If the participant indicate either functional impairment or treatment for depressive or anxiety problems, previous depressive/anxiety problems are coded as present.

Measures of adverse events and exposure severity are not available in English.
